# High Expression of Long Noncoding RNA PCNA-AS1 Promotes Non-Small-Cell Lung Cancer Cell Proliferation and Oncogenic Activity via Upregulating CCND1

**DOI:** 10.7150/jca.39087

**Published:** 2020-01-29

**Authors:** Chuanyong Wu, Xiao-ting Zhu, Lei Xia, Lin Wang, Wenjun Yu, Qiaomei Guo, Mingna Zhao, Jiatao Lou

**Affiliations:** 1 Department of Laboratory Medicine, Shanghai Chest Hospital, Shanghai Jiao Tong University, Shanghai 200030, China.; 2 Department of Laboratory Medicine, Yueyang Hospital of Integrated Traditional Chinese and Western Medicine, Shanghai University of Traditional Chinese Medicine, Shanghai 200437, China.; 3 Department of Anatomy and Physiology, Shanghai Jiao Tong University School of Medicine, Shanghai 200025, China.; 4 Department of orthopedics, Hospital of No.83 army, Xinxiang, 453000, China.

**Keywords:** long noncoding RNA, PCNA-AS1, non-small-cell lung cancer, proliferation, cell cycle, CCND1

## Abstract

Accumulating evidences showed that aberrantly expressed long noncoding RNAs (lncRNAs) have critical roles in many cancers. However, the expression and roles of a poorly studied lncRNA PCNA-AS1 in non-small-cell lung cancer (NSCLC) remain unknown. In this study, we investigated the expression, clinical significance, biological roles, and functional mechanism of PCNA-AS1 in NSCLC. Our results showed that PCNA-AS1 was upregulated in NSCLC tissues and cell lines, and correlated with TNM stages. Functional experiments showed that overexpression of PCNA-AS1 promoted NSCLC cell proliferation and cell cycle progression. Depletion of PCNA-AS1 inhibited NSCLC cell proliferation and cell cycle progression, and also inhibited NSCLC tumor growth *in vivo*. Mechanistically, we found that PCNA-AS1 upregulated CCND1 expression. The expression of PCNA-AS1 was positively correlated with that of CCND1 in NSCLC tissues. Moreover, depletion of CCND1 abrogated the oncogenic roles of PCNA-AS1 in NSCLC. In conclusion, highly expressed PCNA-AS1 promotes NSCLC cell proliferation and oncogenic activity via upregulating CCND1. Our results imply that PCNA-AS1 might serve as a therapeutic target for NSCLC.

## Introduction

Lung cancer is the most common human malignancy and the primary cause of cancer-related death worldwide 1. Non-small-cell lung cancer (NSCLC) accounts for approximate 80% of all lung cancer, which include adenocarcinoma, squamous cell carcinoma, adenosquamous cell carcinoma, and large cell carcinoma 2, 3. Despite great advances in therapeutic methods, including radical resection, chemotherapy, and molecular targeted therapy, the prognosis of NSCLC is still very poor 4, 5. Therefore, it is imperative to elucidate the molecular mechanisms underlying tumorigenesis and progression of NSCLC, and identify novel therapeutic targets for NSCLC.

Long noncoding RNA (lncRNA) is a novel type of noncoding transcript with more than 200 nucleotides in length 6, 7. Accumulating evidences suggest that lncRNAs are involved in various pathophysiological processes, particular in cancers 8-11. Dysregulated expression of lncRNAs have been revealed in several cancers, including lung cancer, hepatocellular carcinoma, gastric cancer, melanoma, and et al. 12-16. LncRNAs also have been found to play critical roles in various cancers, including tumor initiation, progression, and metastasis 17-19. In our previous study, we found that lncRNA GPC3-AS1 is upregulated in hepatocellular carcinoma, associated with poor prognosis, and promotes hepatocellular carcinoma cell proliferation and metastasis 20. The aberrant expressions and roles of several lncRNAs in NSCLC have also been reported, such as PVT1, BCYRN1, MALAT1, HOTAIR 21-24. These studies demonstrated the importance of lncRNAs in cancers, which has previous been recognized as transcriptional noise.

Transcriptome RNA sequencing results revealed that more than 50,000 lncRNAs were identified 25. But the clinical significances and functions of most of these lncRNAs are still unknown. PCNA-AS1 is a recently identified and poorly understood lncRNA, which is reported to be upregulated in hepatocellular carcinoma and promote hepatocellular carcinoma cell growth 26. The gene coding PCNA-AS1 is located at chromosome 20p12.3. PCNA-AS1 has only one exon and is transcribed in antisense orientation with PCNA. However, the expression, clinical significance, and biological roles of PCNA-AS1 in lung cancer are still unknown.

In this study, we measured the expression of PCNA-AS1 in NSCLC tissues and cell lines, and investigated its correlation with clinicopathological characteristics. Using gain-of-function and loss-of-function assays, we explored the biological roles of PCNA-AS1 in NSCLC. Furthermore, the molecular mechanisms underlying the roles of PCNA-AS1 in NSCLC are also explored.

## Materials and Methods

### Tissues samples

Eighty-two NSCLC tissues and paired adjacent non-tumor tissues were excised from patients who were diagnosed with NSCLC and underwent surgery before radiation or chemotherapy at Shanghai Chest Hospital (Shanghai, China). All the tissues were diagnosed by histopathological examination. The tissues were immediately frozen in liquid nitrogen and stored at -80 °C until use. The Research Ethics Committee of Shanghai Chest Hospital approved this study and informed consents were obtained from all patients.

### Cell lines and cell culture

The human normal bronchial epithelial cell line 16HBE and human NSCLC cell lines A549, H460 and H1299 were obtained from the cell bank of the Chinese Academy of Sciences (Shanghai, China). 16HBE cells were cultured in DMEM medium (Invitrogen, Carlsbad, CA, USA). A549, H460 and H1299 cells were cultured in RPMI-1640 medium (Invitrogen). All the medium were supplemented with 10% fetal bovine serum (Invitrogen), and all the cells were maintained in a humidified incubator with 5% CO_2_ at 37 °C.

### RNA isolation and quantitative real-time polymerase chain reaction (qRT-PCR) assays

Total RNA was extracted from tissues or cells with TRIzol reagent (Invitrogen) according to the manufacturer's instruction. First-strand cDNA was generated using the M-MLV Reverse Transcriptase (Invitrogen) and either random primers or gene-specific primers. Quantitative real-time polymerase chain reaction (qRT-PCR) was carried out using the SYBR^®^ Premix Ex Taq™ II kit (Takara, Dalian, China) on ABI StepOnePlus system (Applied Biosystems, Foster City, CA, USA). GAPDH was used as internal normalized reference. The primer sequences used in this study were as follows: PCNA-AS1: 5'-TTGTTGCCACTCCGCCAC-3' (reverse transcription), 5'-TTTGGACATACTGGTGAGG-3' (forward) and 5'-AAGGTGTTGGAGGCACTC-3' (reverse); PCNA: 5'-GCTGTTGTAATTTCCTGTGC-3' (forward) and 5'-CATACTGAGTGTCACCGTTG-3' (reverse); CCND1: 5'-TTCCTGTCCTACTACCGC-3' (forward) and 5'-CTCCTCCTCTTCCTCCTC-3' (reverse); GAPDH: 5'-GGAGCGAGATCCCTCCAAAAT-3' (forward) and 5'-GGCTGTTGTCATACTTCTCATGG-3' (reverse). The relative expression of RNAs was calculated by the comparative Ct method.

### Vectors construction, small interfering RNA (siRNA) synthesis and transfection

PCNA-AS1 full-length transcript was PCR amplified by the TaKaRa Ex Taq^®^ Hot Start Version (Takara) from cDNA which is synthesized from RNA of NSCLC tissues, and subcloned into the Hind III and BamH I sites of pcDNA3.1(+) (Invitrogen), named pcDNA3.1-PCNA-AS1. The primer sequences used were as follows: 5'-CCCAAGCTTCTTCAAATACTAGCGCCAAGGTAT-3' (forward) and 5'-CGGGATCCTTTTTTCGCAACGCGGCGCAGG-3' (reverse). The oligonucleotides for shRNA specifically targeting PCNA-AS1 were synthesized by GenePharma (Shanghai, China). The shRNA target sequence was: 5'-GCTGGAGCTAATATCCCAGCA-3'. After annealing, the oligonucleotides were inserted into the shRNA expression vector pGPU6/Neo (GenePharma). CCND1 specific siRNA was designed and synthesized by Invitrogen. Transfection was carried out using Lipofectamine 3000 (Invitrogen) following the manufacturer's protocol.

### Stable cell lines construction

To constructing PCNA-AS1 stably overexpressed A549 and H1299 cells, pcDNA3.1-PCNA-AS1 or pcDNA3.1 was transfected into A549 and H1299 cells, and selected with neomycin (1000 µg/ml) for four weeks. To constructing PCNA-AS1 stably depleted A549 and H1299 cells, PCNA-AS1 or scrambled shRNAs expression vectors were transfected into A549 and H1299 cells, and selected with neomycin (1000 µg/ml) for four weeks.

### Cell proliferation assays

Cell proliferation was assessed using Cell Counting Kit-8 (CCK-8) assays and Ethynyl deoxyuridine (EdU) incorporation assays. For CCK-8 assays, a total of 3000 cells were plated in each well of 96-well plate. Cell proliferation was measured using the CCK-8 kit (Dojindo, Kumamoto, Japan) at indicated time through collecting the absorbance at 450nm according to the manufacturer's protocol. EdU incorporation assays were performed using the EdU kit (Roche, Mannheim, Germany) following the manufacturer's instruction. The results were collected and quantified using the Zeiss photomicroscope (Carl Zeiss, Oberkochen, Germany) and Image-Pro plus 6.0 software.

### Cell cycle analysis

The cell cycles of indicated NSCLC cells were analyzed using the Cell Cycle Analysis Kit (Beyotime, Jiangsu, China) on FACSCalibur flow cytometer following the manufacturer's instruction.

### Animal experiments

The Research Ethics Committee of Shanghai Chest Hospital approved the animal experiments. Briefly, 3×10^6^ indicted NSCLC cells were subcutaneously injected into five-week-old athymic BALB/c nude mice purchased from the Shanghai Experimental Animal Center of Chinese Academy of Sciences (Shanghai, China). Subcutaneous tumors growth was measured every 7 days with a caliper, and the tumor volume was calculated as 1/2 a×b^2^ (a, long axes; b, short axes).

### Immunohistochemistry assays

Immunohistochemistry was carried out with the standard approach as previously described 27. Briefly, formalin-fixed, paraffin-embedded subcutaneous tumors were cut into 4 µm sections. The sections were incubated with a primary antibody against Ki67 (Abcam, Hong Kong, China). After being washed, the sections were incubated with horseradish peroxidase-conjugated second antibody (Abcam) and visualized using DAB Horseradish Peroxidase Color Development Kit (Beyotime).

### Western blot analysis

Proteins were extracted, quantified and separated by sodium dodecyl sulfate-polyacrylamide gel electrophoresis (SDS-PAGE), and then being transferred to PVDF membrane. After being blocked using 5% not-fat milk, the blots were incubated with antibodies for CCND1 (Abcam) or β-actin (Abcam) at 4 °C overnight. After being washed, the blots were incubated with fluorescence-labeled secondary antibodies, and detected using an Odyssey infrared scanner (Li-Cor, Lincoln, NE, USA).

### Statistical analysis

All data are shown as the mean ± standard deviation (SD) of at least three independent experiments. Comparisons between groups were performed using the SPSS 18.0 software package (SPSS, Chicago, IL, USA) with Wilcoxon signed-rank test, Mann-Whitney test, Student's t-test, Pearson correlation analysis, and one-way ANOVA followed by Dunnett's multiple comparisons test as indicated. *P* values < 0.05 were defined as statistically significant.

## Results

### PCNA-AS1 is upregulated in NSCLC tissues and cell lines

**Figure 1:**
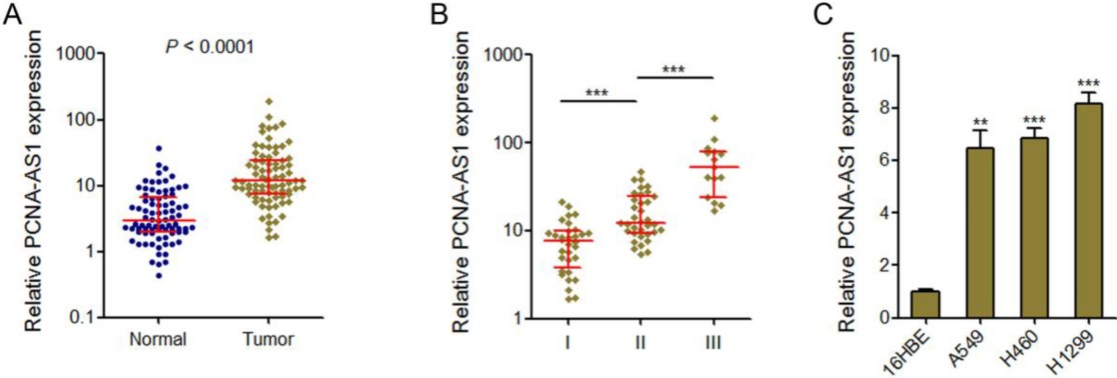
** PCNA-AS1 is highly expressed in NSCLC tissues and cell lines. (A)** PCNA-AS1 expression in 82 pairs of NSCLC tissues and adjacent non-tumor tissues was measured by qRT-PCR. *P* < 0.0001 by Wilcoxon signed-rank test. **(B)** PCNA-AS1 expression in NSCLC tissues with different clinical stages. ***P < 0.001 by Mann-Whitney test. **(C)** PCNA-AS1 expression in normal bronchial epithelial cell line (16HBE) and NSCLC cell lines (A549, H460 and H1299) was measured by qRT-PCR. **P < 0.01, ***P < 0.001 by Student's t-test.

PCNA-AS1 expression levels were measured in 82 pairs of NSCLC tissues and adjacent non-tumor tissues by qRT-PCR assays. The results showed that PCNA-AS1 expression was significantly upregulated in NSCLC tissues compared with adjacent normal tissues (Figure 1A). Next, we investigated the correlation between PCNA-AS1 expression levels and TNM stages. As shown in Figure 1B, PCNA-AS1 expression was significantly upregulated in NSCLC samples with advanced TNM stages. Furthermore, we measured PCNA-AS1 expression in NSCLC cell lines (A549, H460 and H1299) and normal bronchial epithelial cell line (16HBE). As shown in Figure 1C, PCNA-AS1 expression was significantly upregulated in NSCLC cell lines compared with normal bronchial epithelial cell line. Collectively, these results suggested that PCNA-AS1 is upregulated in NSCLC.

### Ectopic expression of PCNA-AS1 promotes NSCLC cell proliferation

**Figure 2:**
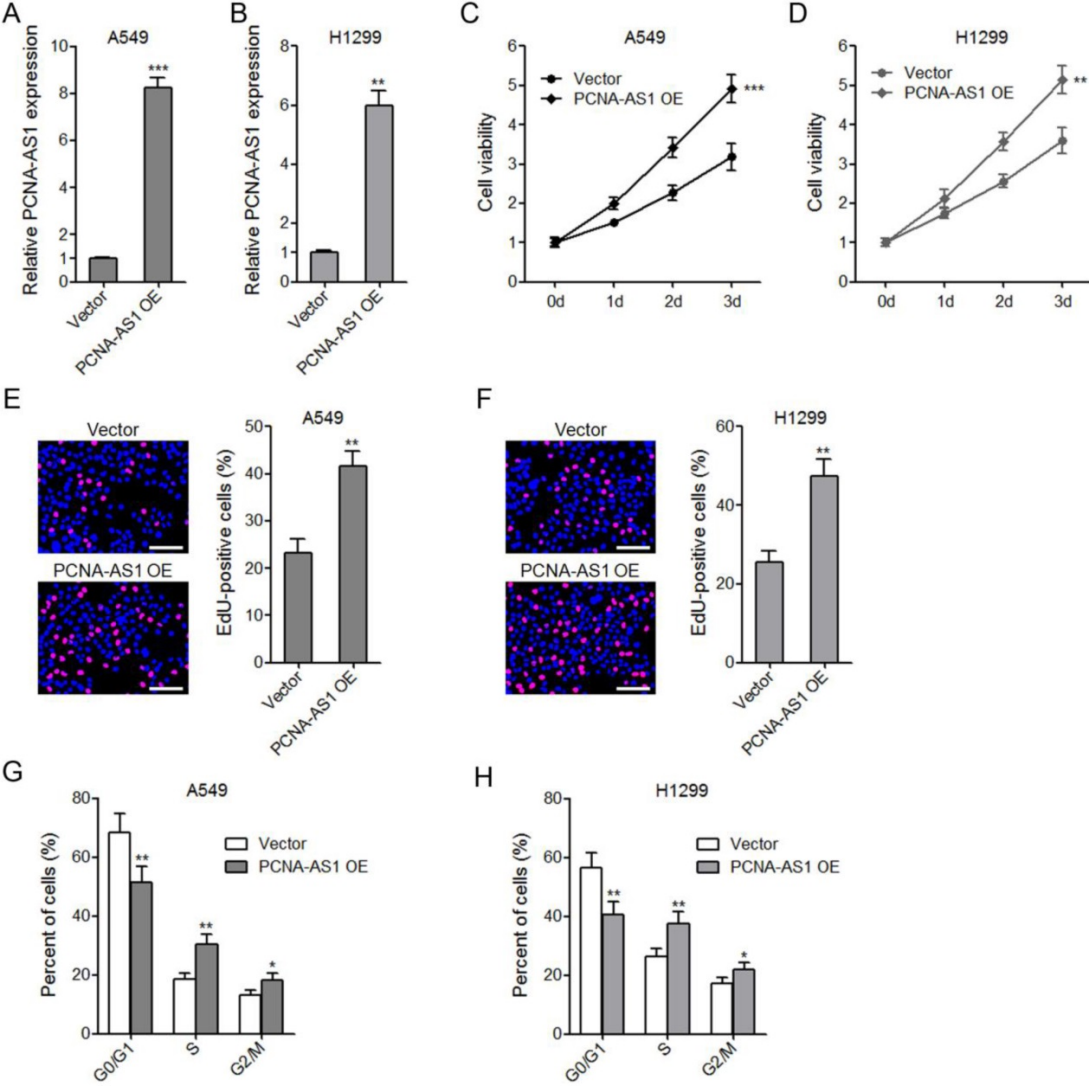
** Ectopic expression of PCNA-AS1 promotes NSCLC cell proliferation and cell cycle progression. (A and B)** PCNA-AS1 expression in PCNA-AS1 stably overexpressed and control A549 and H1299 cells was measured by qRT-PCR. **(C and D)** Cell proliferation of PCNA-AS1 stably overexpressed and control A549 and H1299 cells was detected by CCK-8 assays, and the relative number of cells to 0 day is shown. **(E and F)** Cell proliferation of PCNA-AS1 stably overexpressed and control A549 and H1299 cells was detected by EdU incorporation assays. The red color represents EdU-positive nuclei. Scale bars, 100 μm. **(G and H)** Cell cycle distribution of PCNA-AS1 stably overexpressed and control A549 and H1299 cells was analyzed by flow cytometry. *P < 0.05, **P < 0.01, ***P < 0.001 by Student's t-test.

To explore the biological roles of PCNA-AS1 on NSCLC, we constructed PCNA-AS1 stably overexpressed A549 and H1299 cells (Figure 2A and 2B). CCK-8 assays showed that ectopic expression of PCNA-AS1 promoted A549 and H1299 cell proliferation (Figure 2C and 2D). EdU incorporation assays further verified the pro-proliferative roles of PCNA-AS1 on A549 and H1299 cells (Figure 2E and 2F). Furthermore, flow cytometric analysis revealed that ectopic expression of PCNA-AS1 in both A549 and H1299 cells decreased the percentage of cells at G0/G1 phase (Figure 2G and 2H), implying that PCNA-AS1 promotes NSCLC cell cycle progression.

### Depletion of PCNA-AS1 suppresses NSCLC cell proliferation

**Figure 3:**
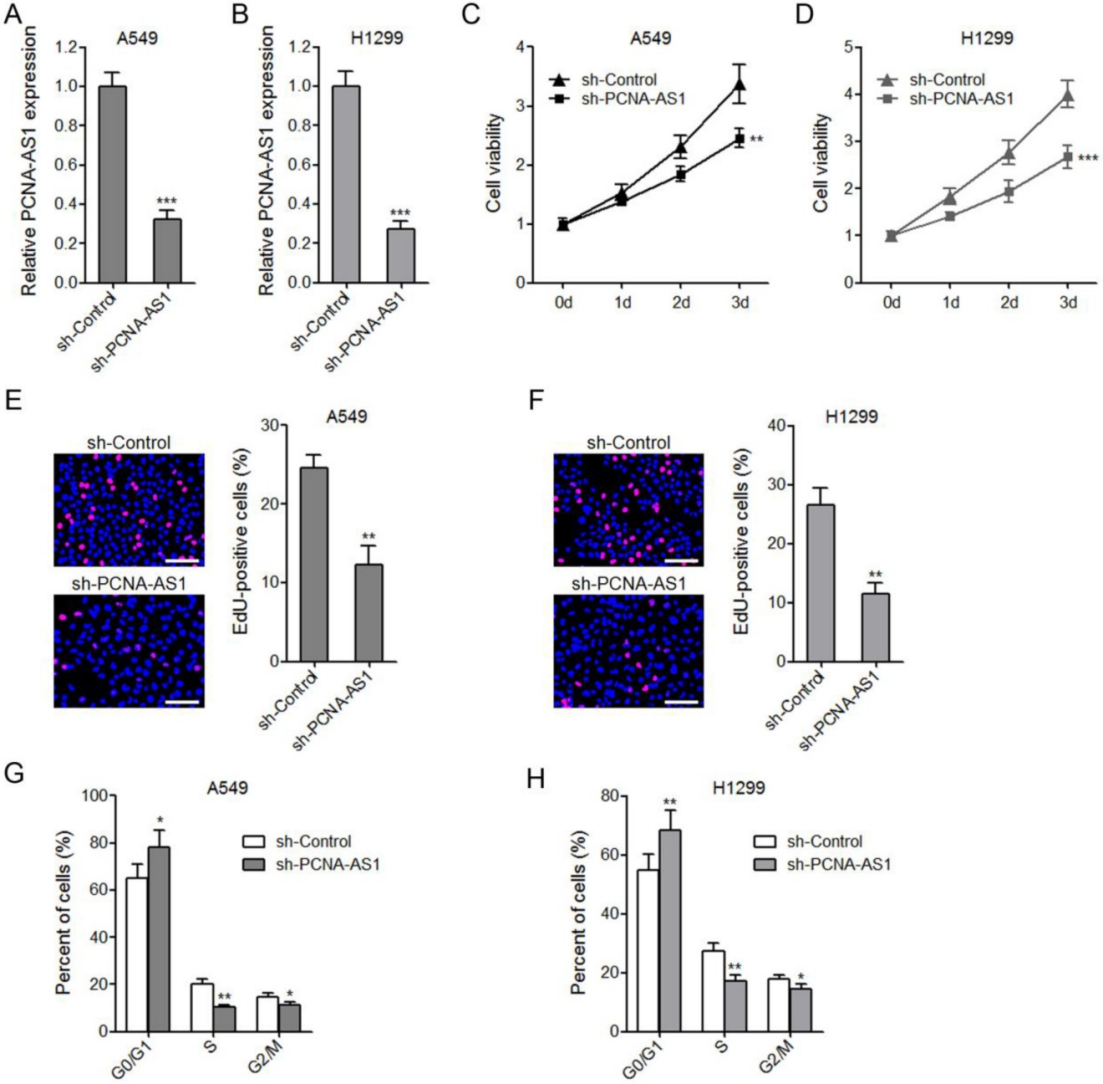
** Depletion of PCNA-AS1 inhibits NSCLC cell proliferation and cell cycle progression. (A and B)** PCNA-AS1 expression in PCNA-AS1 stably depleted and control A549 and H1299 cells was measured by qRT-PCR. **(C and D)** Cell proliferation of PCNA-AS1 stably depleted and control A549 and H1299 cells was detected by CCK-8 assays, and the relative number of cells to 0 day is shown. **(E and F)** Cell proliferation of PCNA-AS1 stably depleted and control A549 and H1299 cells was detected by EdU incorporation assays. The red color represents EdU-positive nuclei. Scale bars, 100 μm. **(G and H)** Cell cycle distribution of PCNA-AS1 stably depleted and control A549 and H1299 cells was analyzed by flow cytometry. *P < 0.05, **P < 0.01, ***P < 0.001 by Student's t-test.

To further confirm the biological roles of PCNA-AS1 on NSCLC, we constructed PCNA-AS1 stably depleted A549 and H1299 cells by transfecting PCNA-AS1-specific shRNA (Figure 3A and 3B). CCK-8 assays showed that depletion of PCNA-AS1 significantly inhibited A549 and H1299 cell proliferation (Figure 3C and 3D). EdU incorporation assays further verified the proliferation inhibitory roles of PCNA-AS1 depletion on A549 and H1299 cells (Figure 3E and 3F). Flow cytometric analysis revealed that depletion of PCNA-AS1 in both A549 and H1299 cells increased the percentage of cells at G0/G1 phase (Figure 3G and 3H), implying that depletion of PCNA-AS1 inhibits NSCLC cell cycle progression.

### Depletion of PCNA-AS1 inhibits NSCLC xenograft tumor growth *in vivo*

**Figure 4:**
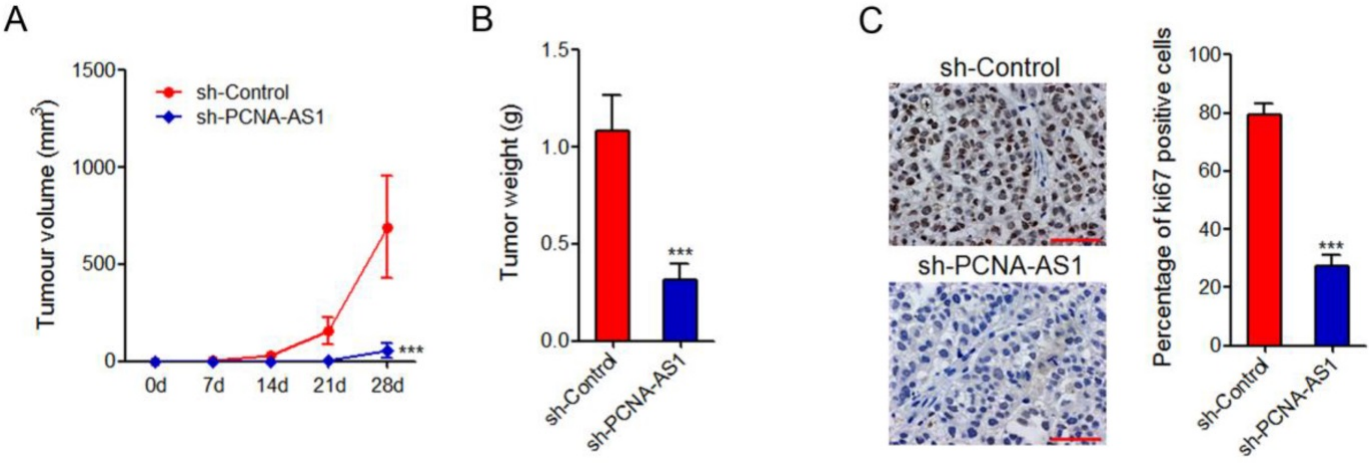
** Depletion of PCNA-AS1 inhibits NSCLC tumor growth *in vivo*. (A)** Tumor volumes of subcutaneous tumors derived from PCNA-AS1 stably depleted and control H1299 cells were measured every 7 days. **(B)** Tumor weights of subcutaneous tumors derived from PCNA-AS1 stably depleted and control H1299 cells at 28 day after injection. **(C)** *In vivo* cell proliferation of PCNA-AS1 stably depleted and control H1299 cells was assessed by Ki67 immunohistochemistry staining of subcutaneous tumors. Scale bars, 50 µm. ***P < 0.001 by Mann-Whitney test.

To further investigate the biological roles of PCNA-AS1 on NSCLC, PCNA-AS1 stably depleted and control H1299 cells were injected subcutaneously into nude mice. Tumor growth rates were measured every 7 days for 28 days, and then the tumors were excised and weighed. The results showed that depletion of PCNA-AS1 significantly inhibited xenograft tumor growth *in vivo* (Figure 4A and 4B). Ki67 staining of xenograft further showed that depletion of PCNA-AS1 inhibited H1299 cell proliferation *in vivo* (Figure 4C).

### PCNA-AS1 upregulates CCND1 expression

**Figure 5:**
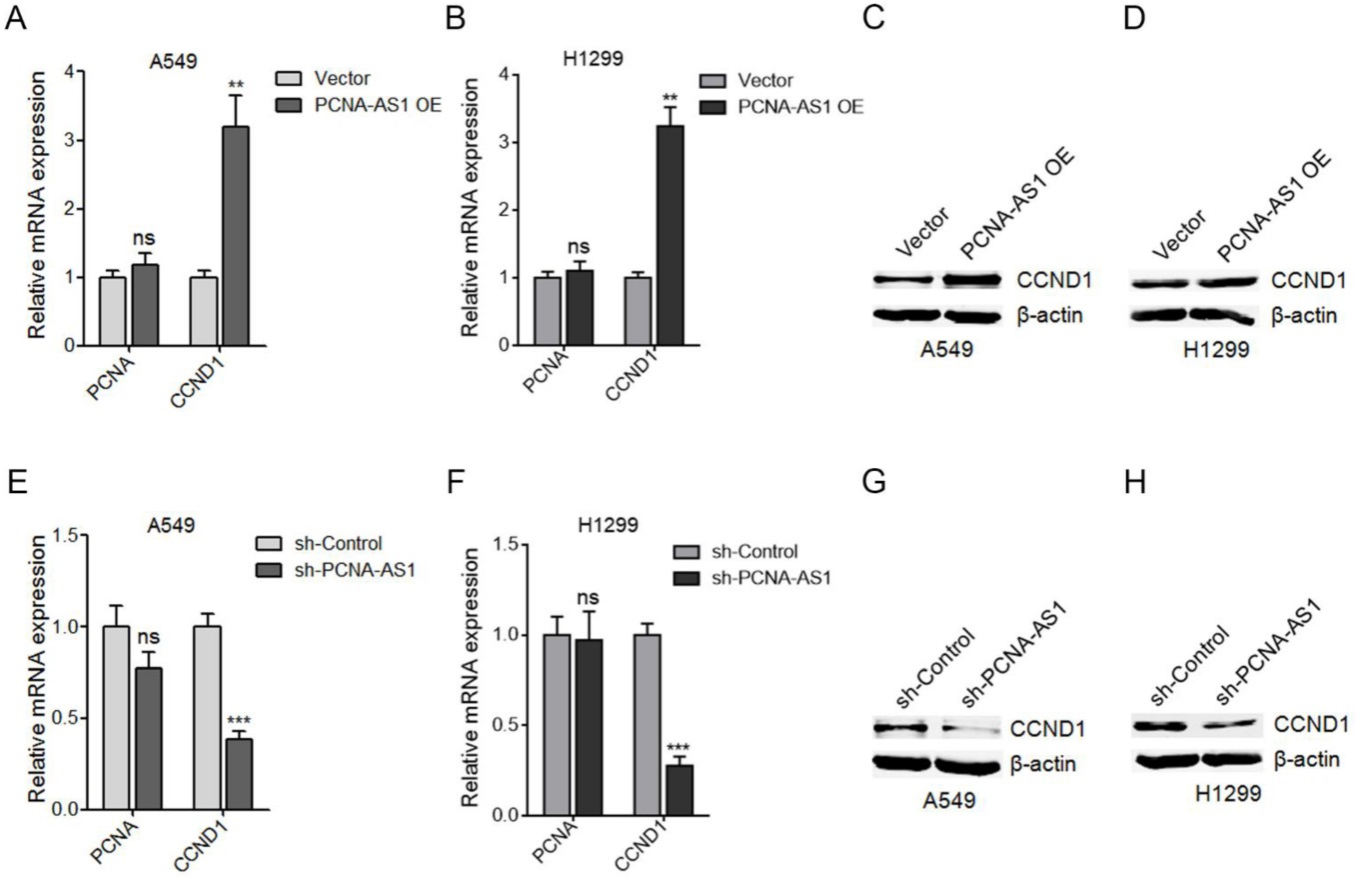
** PCNA-AS1 upregulates CCND1 expression. (A)** PCNA and CCND1 mRNA level in PCNA-AS1 stably overexpressed and control A549 cells was measured by qRT-PCR. **(B)** PCNA and CCND1 mRNA level in PCNA-AS1 stably overexpressed and control H1299 cells was measured by qRT-PCR. **(C and D)** CCND1 protein level in PCNA-AS1 stably overexpressed and control A549 and H1299 cells was measured by western blot. **(E)** PCNA and CCND1 mRNA level in PCNA-AS1 stably depleted and control A549 cells was measured by qRT-PCR. **(F)** PCNA and CCND1 mRNA level in PCNA-AS1 stably depleted and control H1299 cells was measured by qRT-PCR. **(G and H)** CCND1 protein level in PCNA-AS1 stably depleted and control A549 and H1299 cells was measured by western blot. **P < 0.01, ***P < 0.001, ns, not significant, by Student's t-test.

To uncover the molecular mechanisms underlying the pro-proliferative roles of PCNA-AS1 on NSCLC, we first measured the effects of PCNA-AS1 on PCNA, which is transcribed in antisense orientation with PCNA-AS1. However, our results showed that ectopic expression of PCNA-AS1 did not regulate PCNA expression (Figure 5A). Due to the significant effects of PCNA-AS1 on G0/G1 phase cell cycle arrest, we next detected the effects of PCNA-AS1 on CCND1 (cyclin D1), whose activity is required for G1/S transition 28. As shown in Figure 5A-D, ectopic expression of PCNA-AS1 significantly upregulated CCND1 mRNA and protein levels in both A549 and H1299 cells. In addition, although depletion of PCNA-AS1 did not regulate PCNA expression, depletion of PCNA-AS1 significantly decreased CCND1 mRNA and protein levels in both A549 and H1299 cells (Figure 5E-H). These results suggested that PCNA-AS1 upregulates CCND1 expression in NSCLC cells.

### PCNA-AS1 transcript level was positively correlated with CCND1 mRNA level in NSCLC tissues

**Figure 6:**
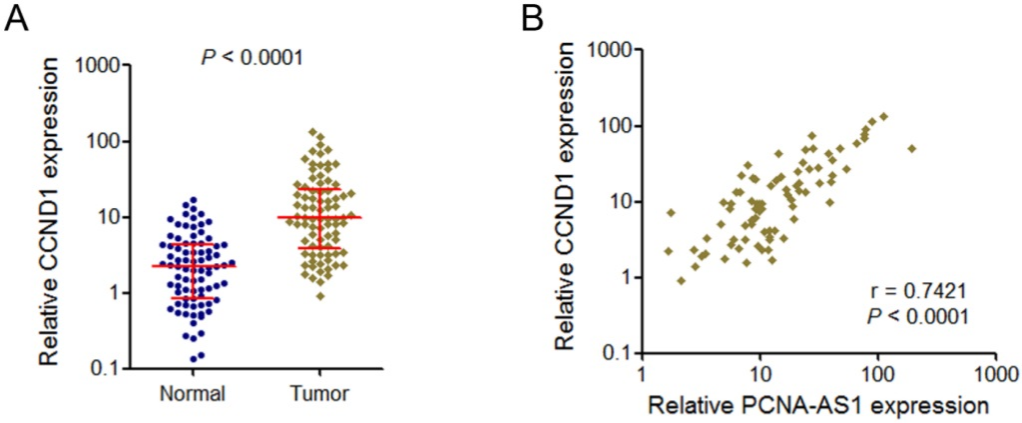
** PCNA-AS1 transcript level is positively correlated with CCND1 mRNA level in NSCLC tissues. (A)** CCND1 mRNA level in 82 pairs of NSCLC tissues and adjacent non-tumor tissues was measured by qRT-PCR. *P* < 0.0001 by Wilcoxon signed-rank test. **(B)** The correlation between PCNA-AS1 transcript level and CCND1 mRNA level was measured in 82 NSCLC tissues. *P* < 0.0001 by Pearson correlation analysis.

Because PCNA-AS1 could upregulate CCND1 expression, we next investigate whether a correlation exists between PCNA-AS1expression level and CCND1 in human NSCLC tissues. We examined CCND1 mRNA level in the same set of 82 pairs of NSCLC tissues and adjacent normal tissues shown in Figure 1A. The results showed that CCND1 is also significantly upregulated in NSCLC tissues compared with adjacent normal tissues (Figure 6A). Correlation analysis showed that PCNA-AS1 expression level was positively correlated with CCND1 mRNA level in these 82 NSCLC tissues (Figure 6B).

### Depletion of CCND1 abolished the pro-proliferative roles of PCNA-AS1 on NSCLC cells

**Figure 7:**
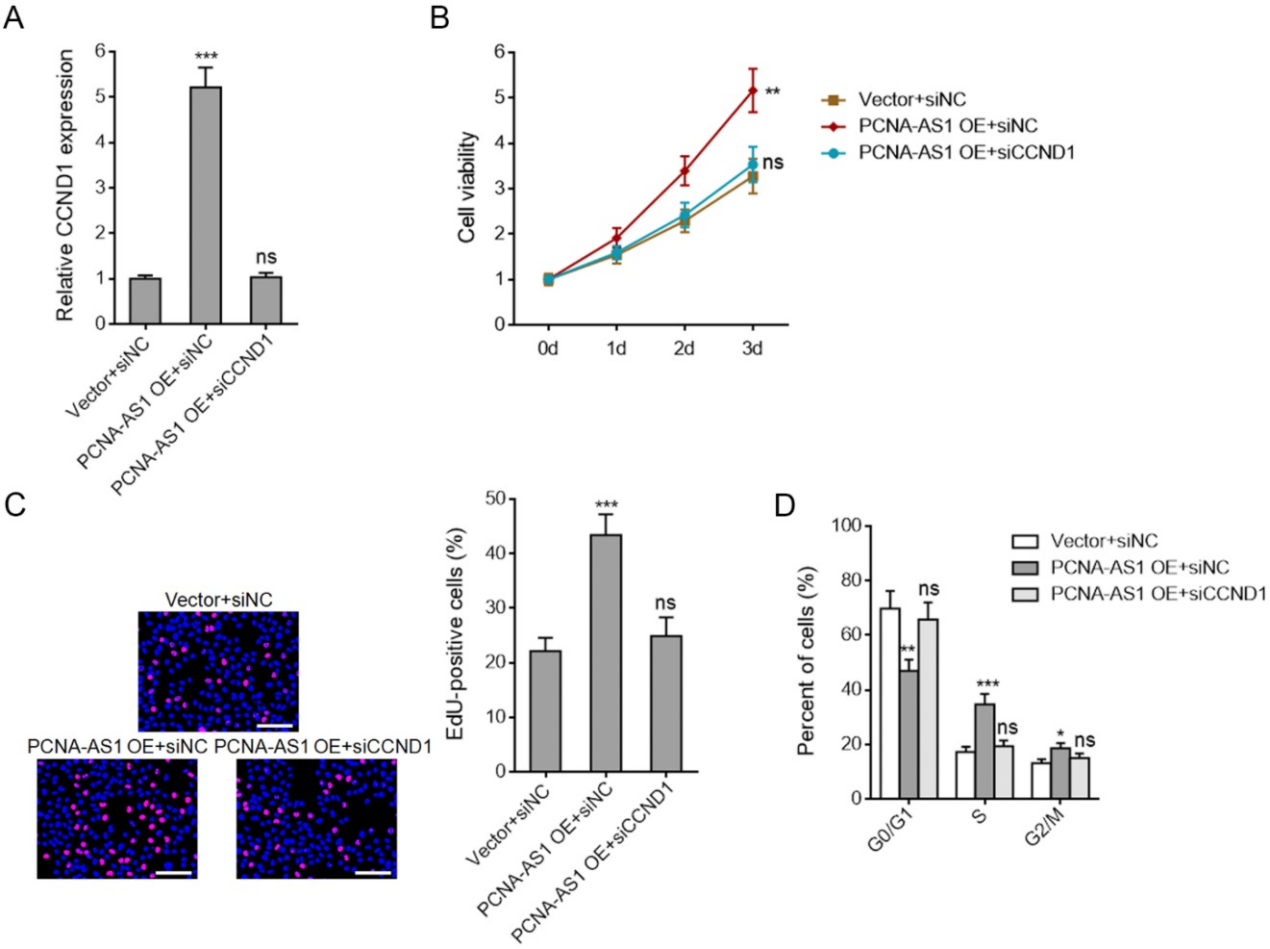
** Depletion of CCND1 abrogates the biological roles of PCNA-AS1 on NSCLC cell proliferation and cell cycle progression. (A)** CCND1 expression in PCNA-AS1 stably overexpressed and control A549 cells after transfection with CCND1 specific siRNA was measured by qRT-PCR. **(B)** Cell proliferation of PCNA-AS1 stably overexpressed and control A549 cells after transfection with CCND1 specific siRNA was detected by CCK-8 assays, and the relative number of cells to 0 day is shown. **(C)** Cell proliferation of PCNA-AS1 stably overexpressed and control A549 cells after transfection with CCND1 specific siRNA was detected by EdU incorporation assays. The red color represents EdU-positive nuclei. Scale bars, 100 μm. **(D)** Cell cycle distribution of PCNA-AS1 stably overexpressed and control A549 cells after transfection with CCND1 specific siRNA was analyzed by flow cytometry. *P < 0.05, **P < 0.01, ***P < 0.001, ns, not significant, by one-way ANOVA followed by Dunnett's multiple comparisons test.

To investigate whether PCNA-AS1 promotes NSCLC cell proliferation through upregulating CCND1 expression, we silenced CCND1 expression in PCNA-AS1 stably overexpressed A549 cells (Figure 7A). CCK-8 assays showed that CCND1 depletion abolished the pro-proliferative roles of PCNA-AS1 on A549 cells (Figure 7B). EdU incorporation assays further verified that CCND1 depletion could abolish the pro-proliferative roles of PCNA-AS1 on A549 cells (Figure 7C). Flow cytometric analysis revealed that CCND1 depletion reversed the down-regulation of percentage of cells at G0/G1 phase caused by PCNA-AS1 overexpression (Figure 7D), implying that CCND1 depletion reversed NSCLC cell cycle progression caused by PCNA-AS1 overexpression. Collectively, these results suggested that the roles of PCNA-AS1 on NSCLC cell proliferation and cell cycle are dependent on CCND1.

## Discussion

With the advances of oncological researches, many critical oncogenes or tumor suppressors in NSCLC were identified, such as p53, EGFR, PD-1/PD-L1 29-31. Several molecular targeted therapies have been developed, such as EGFR-tyrosine kinase inhibitor and PD-1/PD-L1 antibodies 32, 33. However, many NSCLC patients are not sensitive to these therapies 34. This also implies that the carcinogenesis and progression of NSCLC are very complex and many genes participate in these processes.

Recently, the crucial roles of lncRNAs in cancers have been revealed 35. MALAT1, which is a well-known lncRNA, promotes lung cancer metastasis 23. PVT1, another well studied lncRNA, promotes NSCLC tumorigenesis and cell proliferation 21. Several transcriptome RNA sequencing results demonstrated that the number of lncRNAs is very larger than that of mRNAs, and over 68% of all transcripts could be classified as lncRNAs 25. Among these many lncRNAs, only a few of them has been studied. Many other lncRNAs may also have important roles in cancers. In this study, we investigated the expression and functions of a previously poorly understood lncRNA PCNA-AS1 in NSCLC. Our results showed that PCNA-AS1 is upregulated in NSCLC tissues and cell lines and positively associated with advanced TNM stages. Gain-of-function experiments showed that overexpression of PCNA-AS1 promotes NSCLC cell proliferation and cell cycle progression. Loss-of-function experiments showed that depletion of PCNA-AS1 inhibits NSCLC cell proliferation, cell cycle progression, and *in vivo* tumor growth. These data demonstrate that the upregulated PCNA-AS1 functions as an oncogene in NSCLC and targeting PCNA-AS1 may be a potential therapeutic strategy for NSCLC.

PCNA-AS1 is oriented in antisense direction with respect to PCNA, which has critical roles in DNA replication and cell proliferation 36. Several antisense lncRNAs are reported to regulate the genes of the opposite strand 20, 37. In this study, we also investigated the effects of PCNA-AS1 on PCNA. However, our results showed that neither overexpression nor depletion of PCNA-AS1 changed PCNA expression. As PCNA-AS1 had significant effects on G0/G1 phase cell cycle arrest, we investigated the regulation of PCNA-AS1 on CCND1 (cyclin D1), which is crucial for G1/S transition. Our results showed that overexpression of PCNA-AS1 upregulates CCND1 expression, and while depletion of PCNA-AS1 decreases CCND1 expression. Moreover, the expression of PCNA-AS1 is positively associated with that of CCND1 in clinical NSCLC tissues, supporting the regulation of CCND1 by PCNA-AS1. Functional experiments showed that depletion of CCND1 abolished the pro-oncogenic roles of PCNA-AS1 on NSCLC. These data suggest that PCNA-AS1 promotes NSCLC cell proliferation and oncogenic activity via upregulating CCND1. Some nuclear lncRNAs could epigenetically modulate the expression of their target genes via binding and recruiting DNA or histone modification enzymes. Some cytoplasmic lncRNAs could directly bind proteins, mRNAs, and/or microRNAs, and further change the location, expression, roles of the interacted partners. The potential molecular mechanism underlying the regulation of CCND1 by PCNA-AS1 needs further investigation.

In conclusion, we found that PCNA-AS1 is increased in NSCLC, promotes NSCLC cell proliferation and oncogenic activity via upregulating CCND1. Our results suggest that PCNA-AS1 would be a potential therapeutic target for NSCLC.
